# Synthetic Cathinones: Recent Developments, Enantioselectivity Studies and Enantioseparation Methods

**DOI:** 10.3390/molecules27072057

**Published:** 2022-03-22

**Authors:** Ana Sofia Almeida, Bárbara Silva, Paula Guedes de Pinho, Fernando Remião, Carla Fernandes

**Affiliations:** 1 Laboratório de Química Orgânica e Farmacêutica, Departamento de Ciências Químicas, Faculdade de Farmácia, Universidade do Porto, Rua Jorge Viterbo Ferreira nº 228, 4050-313 Porto, Portugal; anasofiaalmeida1998@gmail.com (A.S.A.); barbarapolerisilva@gmail.com (B.S.); 2 UCIBIO—Applied Molecular Biosciences Unit, REQUIMTE, Laboratory of Toxicology, Department of Biological Sciences, Faculty of Pharmacy, University of Porto, Rua de Jorge Viterbo Ferreira nº 228, 4050-313 Porto, Portugal; pguedes@ff.up.pt (P.G.d.P.); remiao@ff.up.pt (F.R.); 3 Centro Interdisciplinar de Investigação Marinha e Ambiental (CIIMAR), Universidade do Porto, Terminal de Cruzeiros do Porto de Leixões, Avenida General Norton de Matos, s/n, 4450-208 Matosinhos, Portugal; 4 Associate Laboratory i4HB—Institute for Health and Bioeconomy, Faculty of Pharmacy, University of Porto, 4050-313 Porto, Portugal

**Keywords:** synthetic cathinones, chirality, enantioselectivity, enantiomeric resolution

## Abstract

New psychoactive substances represent a public health threat since they are not controlled by international conventions, are easily accessible online and are sold as a legal alternative to illicit drugs. Among them, synthetic cathinones are widely abused due to their stimulant and hallucinogenic effects. To circumvent the law, new derivatives are clandestinely synthesized and, therefore, synthetic cathinones keep emerging on the drug market, with their chemical and toxicological properties still unknown. In this review, a literature assessment about synthetic cathinones is presented focusing on the recent developments, which include more than 50 derivatives since 2014. A summary of their toxicokinetic and toxicodynamic properties are also presented. Furthermore, synthetic cathinones are chiral compounds, meaning that they can exist as two enantiomeric forms which may present different biological and toxicological activities. To analyze the enantiomers, the development of enantiomeric resolution methods for synthetic cathinones is crucial. Many methods have been reported over the years that include mostly chromatographic and electromigration techniques, with liquid chromatography using chiral stationary phases being the technique of choice. This review intended to present an overview of enantioselectivity studies and enantioseparation analysis regarding synthetic cathinones, highlighting the relevance of chirality and current trends.

## 1. Introduction

The use of new psychoactive substances (NPS) has been growing since 2000 [1]. These substances started to replace illicit drugs as legal alternatives being known as “legal highs”, “smart drugs” or “research chemicals” [2,3]. They can be sold, as bath salts, plant fertilizers or air fresheners. Although these products are frequently labeled as “not for human consumption”, they are mostly purchased with that purpose. Therefore, NPS are defined as new narcotics or psychotropic substances, in pure form or in mixture preparations, that are not controlled by international conventions but can represent a public health concern [3,4].

**Figure 1:**
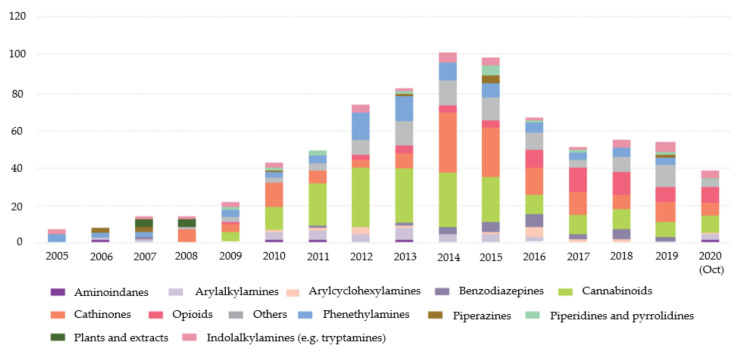
New psychoactive substances reported for the first time from 2005 to 2020 (October) divided by categories [5].

Between 1997 and 2020, the European Monitoring Centre for Drugs and Drug Addiction (EMCDDA) was monitoring more than 820 NPS. Despite this number, it is possible to observe that, since 2015, the number of NPS notified for the first time has been decreasing (Figure 1). In 2019, 53 NPS were reported for the first time and in 2020, until October, the number was 38 [5].

The popularity of these drugs had a sharp increase due to their easy accessibility online. Human and animal studies involving these NPS are very limited and non-existent for some of them. Therefore, available information about the pharmacological and toxicological properties of these substances is still very limited. The actual composition of NPS sold online can be very different from the package label and, therefore, consumers might purchase and use them mistakenly. All these factors explain why the world of new psychoactive substances represents a huge danger for public health [3,4].

**Figure 2:**
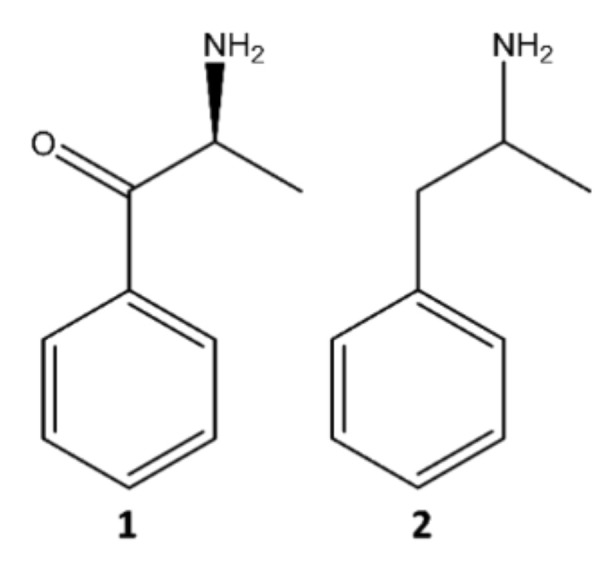
Structures of cathinone (**1**) and amphetamine (**2**).

The two groups of NPS reported on a larger scale are synthetic cannabinoids and synthetic cathinones, representing more than two-thirds of all available compounds since 2005 [5,6]. The present paper will focus on synthetic cathinones, which comprise a vast group of compounds derived from cathinone (**1**), an alkaloid found in khat (*Catha edulis*) leaves, structurally identical and similar in action to amphetamine (**2**) (Figure 2) [7]. 

**Figure 3:**
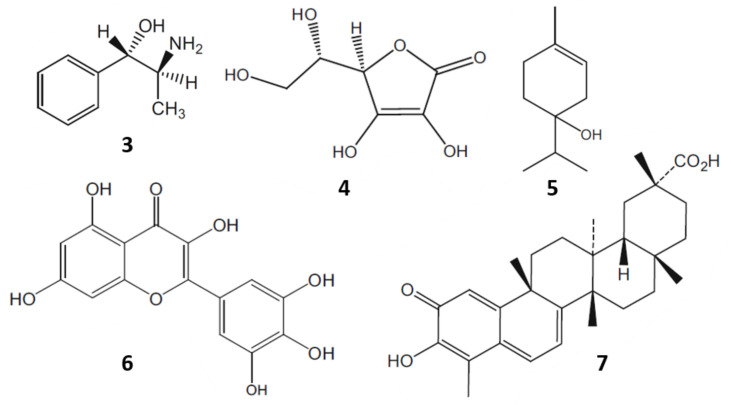
Some bioactive components of khat: cathine (**3**); ascorbic acid (**4**); α-terpineol (**5**); myricetin (**6**); and celastrol (**7**).

Moreover, chewing of fresh khat leaves has been a tradition for centuries in some cultures. The leaves contain more than 40 components such as alkaloids, flavonoids, amino acids, glycosides, sterols, vitamins and minerals (Figure 3) [8]. In 1930, cathine or (+)-norpseudoephedrine (**3**) was identified as the active principle of khat. However, the activity of cathine (**3**) was insufficient to be responsible for all the pharmacological effects observed. Later on, in 1975, cathinone was isolated and found to be seven to ten times more bioactive than cathine (**3**) [6]. 

Although both cathine and cathinone are internationally controlled, the World Health Organization considered that the evidence was insufficient to justify the international control of khat. Nonetheless, they advised the development of campaigns to educate the public about the potential adverse effects of the excessive use of khat [9]. Khat has been used as starting material to synthesize derivatives resulting in the first synthetic cathinones that, in the 2000s, emerged in the drug market [6]. Synthetic cathinones gradually became available in smartshops, internet and other drug paraphernalia stores [10]. They were commonly found as “bath salts” under names like Bloom, Ivory Wave, Vanilla Sky, Blue Silk, or Purple Wave [7]. By 2013, approximately 600 “dark web” sites were identified in Europe that allowed the purchase of these compounds anonymously using untraceable digital currencies [11].

Synthetic cathinones are widely abused due to their stimulant and hallucinogenic effects, replacing 3,4-methylenedioxymethamphetamine (MDMA), cocaine and amphetamines, which are much more expensive. However, synthetic cathinones can be much more potent than the drugs that they are intended to mimic, increasing the risk of overdose and death [12]. 

In the literature, some fundamental reviews about the development, pharmacokinetics, mechanisms of action, and biological/toxicological effects of synthetic cathinones can be found [6,13,14,15]. One of the main objectives of this review is the reporting of new synthetic cathinones that have been clandestinely synthesized and emerged on the drug market in the last few years, to infer about current trends. Special focus is given to stereochemistry issues for this class of compounds which, in many studies, is still not considered. A compilation of enantioselectivity studies as well as enantioseparation analysis of synthetic cathinones is also presented, highlighting the relevance of chirality.

## 2. Classification of Synthetic Cathinones

**Figure 4:**
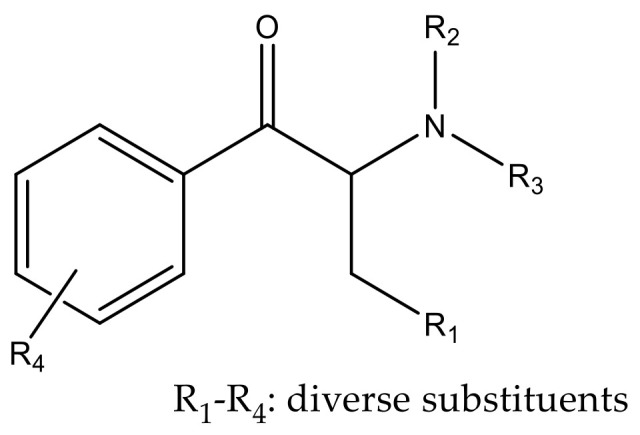
Core structure of cathinone derivatives.

Synthetic cathinones are β-keto phenethylamine derivatives presenting the same core structure. Moreover, they are structurally similar to amphetamine, with the difference being the presence of a keto group [14,16]. Cathinone derivatives can be synthesized by the addition of several substituents at different sites of the cathinone scaffold as represented in Figure 4 [17].

**Figure 5:**
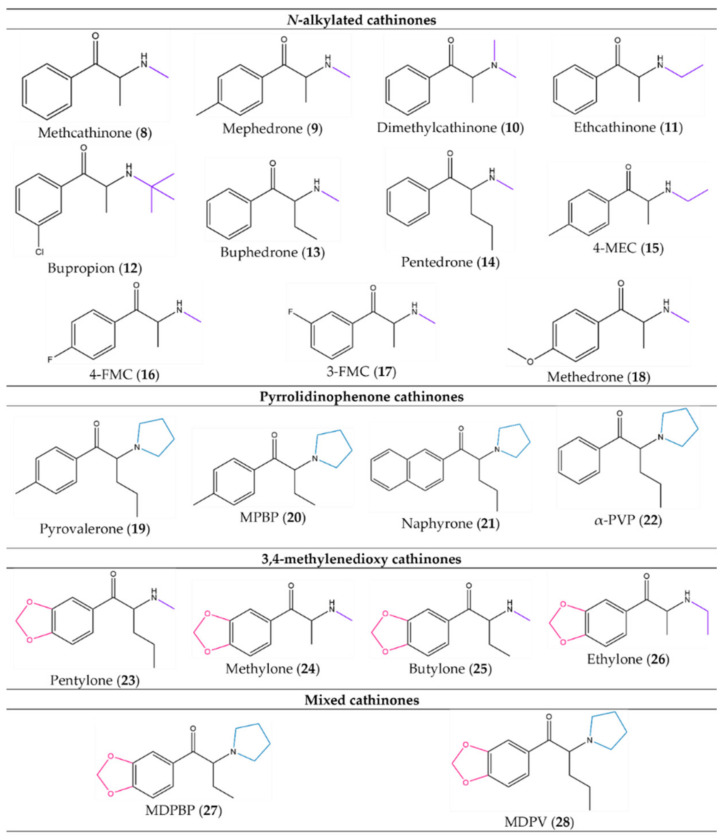
Examples of synthetic cathinones from each group based on the substitution pattern: (**8**)–(**18**) from the *N*-alkylated cathinones, (**19**)–(**22**) from the pyrrolidinophenone cathinones, (**23**)–(**26**) from the 3,4-methylenedioxy cathinones and (**27**) and (**28**) from the mixed cathinones.

Consequently, synthetic cathinones can be divided into four groups based on the substitution pattern (Figure 5). The first group, the *N*-alkylated cathinones (**8–18**), which includes methcathinone (**8**), mephedrone (**9**), dimethylcathinone (**10**) and ethcathinone (**11**), are *N*-substituted compounds with an unsubstituted or substituted phenyl ring. Most of the first synthetic cathinones are part of this group. Pyrrolidinophenone derivatives, the second group, contain in their structure a substituted or unsubstituted phenyl ring and a pyrrolidinyl ring in the side chain. Pyrovalerone (**19**), MPBP (**20**), naphyrone (**21**) and α-PVP (**22**) are examples. The third group are the 3,4-methylenedioxy cathinones to which belong pentylone (**23**), methylone (**24**), butylone (**25**) and ethylone (**26**). They are characterized by a 1,3-benzodioxol-5-yl ring and a straight side chain. Moreover, they present similar structure and pharmacological properties to MDMA. Lastly, the fourth group comprises mixed cathinones or 3,4-methylenedioxypyrrolidinophenones, for instance, MDPBP (**27**) and MDPV (**28**), which present in their structure a combination of the last two groups’ moieties: a methylenedioxyl ring and a pyrrolidinyl side ring [14,16].

## 3. Chronological Evolution and Recent Developments of Cathinone Derivatives 

**Figure 6:**
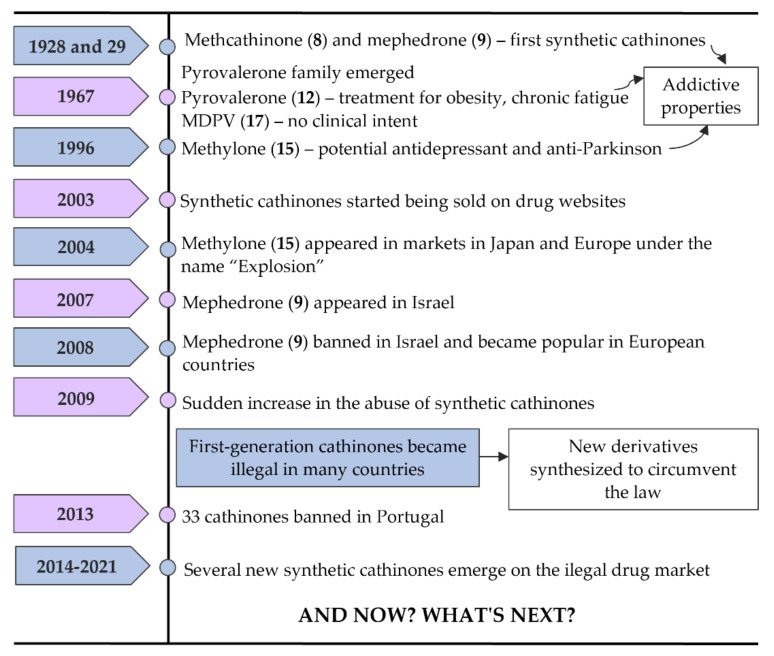
Timeline of events related to the history of synthetic cathinones.

In Figure 6, some of the most important landmarks of the history of synthetic cathinones are summarized.

Methcathinone (α-methylamino-propiophenone or ephedrone (EPH), (**8**)) and mephedrone (4-methylmethcathinone or MEPH, (**9**)) (Figure 5) were the first synthetic cathinones, arising in 1928 and 1929, respectively [2]. Methcathinone (**8**) was meant to reach the market as an antidepressant, but latter it was found to have powerful addictive properties [6,18]. As a consequence, this synthetic derivative was responsible for several intoxications in the Soviet Union in the 70s and in the USA in the 90s being known in the streets as “Cat”, “Jeff” and “Mulka” [13].

The pyrrolidinophenone family comprises a range of compounds that began to be reported at the end of the 60s. Pyrovalerone (**19**, Figure 5) is a member of this family and was firstly synthesized as a treatment for obesity, chronic fatigue and lethargy but, due to its addictive potential, the clinical use was stopped after these reports of abuse [6]. However, other derivatives of this family, such as 3,4-methylenedioxypyrovalerone (MDPV (**28**), Figure 5) in 1967, were synthetized with no clinical intent [2,6]. 

In 1996, methylone (3,4-methylenedioxy-*N*-methylcathinone or ßk-MDMA, (**24**) appeared as a potential antidepressant and anti-Parkinson agent but this compound never reached the market due to their psychostimulant properties identical to MDMA [6,19]. 

From the few cathinones synthetized with a medicinal intent, only bupropion (**12**) succeeded for that purpose, being currently used as an antidepressant and a support to smoking cessation [13,20]. 

Synthetic cathinones had barely any attention until 2003 when they were first reported online on drug websites as a legal replacement to MDMA [7].

Around 2004, methylone (**24**) started to appear in markets in Japan and Europe under the name “Explosion”, also being the first of these substances to be sold via smartshops and online [13]. In 2007, mephedrone (**9**) made its appearance on the market, first in Israel, although it was banned in this country in 2008. After this, mephedrone (**9**), also known in the markets as “Meph”, “TopCat”, “Mcat”, “Meow Meow”, among other names, became more popular in European countries [6,13]. Many drug users began to replace cocaine and ecstasy with mephedrone (**9**) due to a decrease in the purity and availability of the first two drugs. Additionally, mephedrone (**9**) was less expensive and more potent. This explains why, in 2009, there was a sudden increase in the abuse of synthetic cathinones, especially mephedrone (**9**) [13].

Later, this first-generation of cathinones became illegal in many countries. To overcome this, clandestine chemists started to modify their structures to obtain new derivatives that could circumvent the law. Thus, several new cathinones were synthetized such as buphedrone (**13**), butylone (**25**), ethylone (**26**), pentedrone (**14**) and its constitutional isomer 4-methyl-*N*-ethylcathinone (4-MEC, (**15**)). Additionally, flephedrone (4-fluoromethcathinone or 4-FMC (**16**)) and 3-fluoromethcathinone (3-FMC (**17**)), two derivatives of mephedrone (**9**) were also reported [14].

Naphyrone (naphthylpyrovalerone (**21**)), which appeared after mephedrone (**9**) was marketed in the UK under the name “Energy-1” (NRG-1) as a legal alternative [13]. Simultaneously with pentedrone (**14**), a derivative from the same group, α-pyrrolidinopentiophenone (α-PVP, (**22**)) also emerged [14]. The popularity of this compound increased greatly in Europe and the USA between 2011 and 2015, being found in the markets as “Flakka” or “Gravel”. After several fatal or almost-fatal cases of abuse, α-PVP (**22**) began to be controlled internationally [13]. Moreover, in Portugal, in April of 2013, because of new legislative control measures, the commercialization and use of 33 cathinone derivatives were prohibited and all smartshops were closed [6,21].

**Figure 7:**
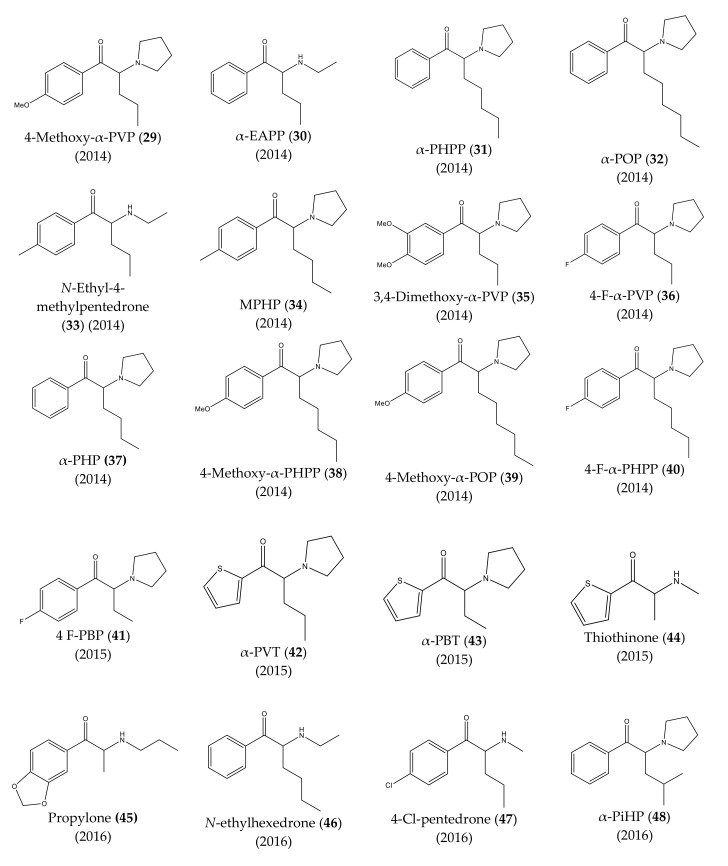
Structures of the most recent cathinone derivatives (**29**)–(**48**).

**Figure 8:**
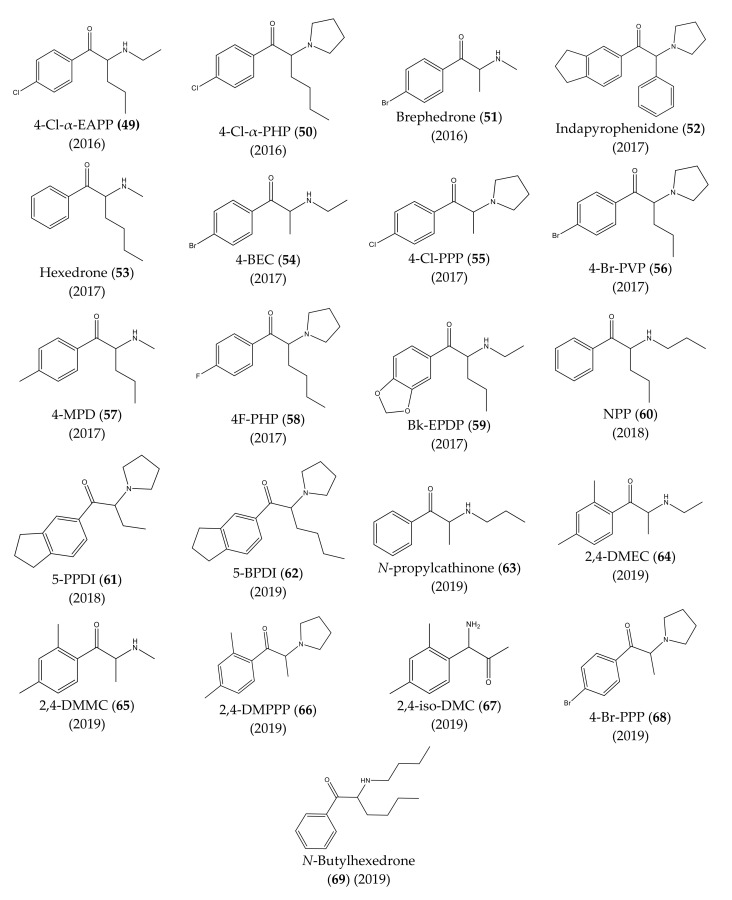
Structures of the most recent cathinone derivatives (**49**)–(**69**).

The chemical structures of the first cathinones are being constantly modified. Each year several new derivatives emerge on the illegal drug market. The chemical structures of synthetic cathinones (**29**–**69**) clandestinely synthetized and reported since 2014 are represented in Figure 7 and Figure 8.

In 2014, several new synthetic cathinones were found in the Japanese market, being sold as “aroma liquids” and “fragrance powders”. They included 4-methoxy-α-PVP (**29**), α-EAPP (**30**), α-PHPP or PV8 (**31**), α-POP or PV9 (**32**), *N*-ethyl-4-methylpentedrone (**33**), MPHP (**34**), 3,4-dimethoxy-α-PVP (**35**), 4-F-α-PVP (**36**). Almost half a year later, α-PHP (**37**), 4-methoxy-α-PHPP (**38**), 4-methoxy-α-POP (**39**), and 4-F-α-PHPP (**40**) were also discovered [13,14,22,23,24]. 

Moreover, in Portugal, 4-F-PBP (**41**) was reported for the first time in 2015 [13,25]. In the same year, the first thienyl cathinone derivatives, α-PVT (**42**), α-PBT (**43**) and bromothienyl analogs were identified [14,26]. The thiothinone (**44**), another thienyl cathinone derivative was also discovered, at the same time [14,27].

In 2016, propylone (**45**), *N*-ethylhexedrone (**46**), 4-chloro-pentedrone (**47**), α-PiHP (**48**), 4-Cl-α-EAPP (**49**) and 4-Cl-α-PHP (**50**) were identified for the first time [13,28]. In the same year, brephedrone (**51**) was identified in seized samples from Brazilian streets. However, this synthetic cathinone had already been reported in other countries [29]. 

In 2017, an unknown compound found in seized drugs in the UK was identified and characterized as indapyrophenidone (**52**), a novel cathinone derivative [30]. Additionally, hexedrone (**53**), 4-BEC (**54**), 4-Cl-PPP (**55**) and 4-Br-PVP (**56**) were first reported in Poland [13,31]. Furthermore, in the same year, three emerging cathinone derivatives: 4-MPD (**57**), 4F-PHP (**58**) and bk-EPDP (**59**) were detected, identified, and fully characterized [32]. 

In 2018, 5-PPDI (**61**), a novel synthetic cathinone, was identified and characterized in an unknown white powder [33]. 

One year later, seven other new synthetic cathinones were reported in Poland: 5-BPDI (**62**), *N*-propylcathinone (**63**), 2,4-DMEC (**64**), 2,4-DMMC (**65**), 2,4-DMPPP (**66**), 2,4-iso-DMC (**67**) and 4-Br-PPP (**68**) [34]. Additionally, in the same year, *N*-butylhexedrone (**69**) was identified in seized material [35], which was, later on, characterized by spectroscopic and crystallographic analysis [36]. 

**Figure 9:**
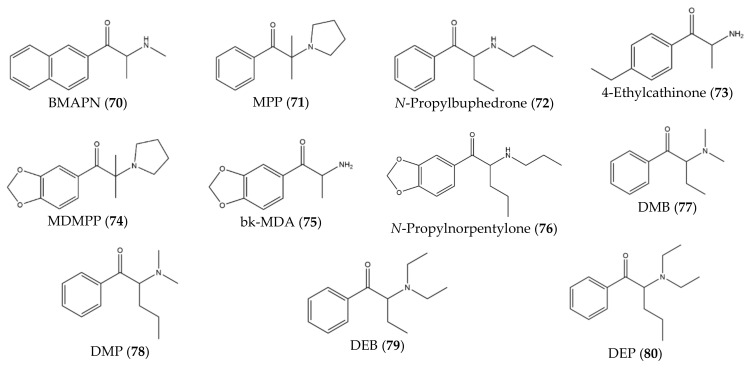
Structures of cathinone derivatives synthesized in controlled laboratories (**70**)–(**80**).

Besides that, some studies have described novel synthetic cathinones (**70–80**) that were synthesized in controlled laboratories (instead of found in the drug market) with the purpose of studying potential effects and to develop analytical techniques for the identification and characterization of future cathinones (Figure 9). 

For instance, Botanas et al. [37] (2017) synthesized a novel synthetic cathinone, BMAPN (**70**), with the purpose of studying its rewarding and reinforcing properties. Since this compound presents a naphthalene substituent on the aromatic ring, this study can be helpful to predict the abuse potential of future cathinones with aromatic ring substitutions [37]. Moreover, Carlsson et al. [38] (2018) synthesized six novel synthetic cathinones: MPP, **71**), *N*-propylbuphedrone (**72**), 4-ethylcathinone (**73**), MDMPP (**74**), bk-MDA (**75**), *N*-propylnorpentylone (**76**). This study described the synthesis of these analogs and provided spectroscopic data [38]. With the same purpose, Gaspar et al. [39] synthesized four novel synthetic cathinones: DMB (**77**), DMP (**78**), DEB (**79**) and DEP (**80**).

Some of the new cathinone derivatives have been described in cases of abuse. For instance, Hasegawa et al. [40] (2014) reported a fatal poisoning case of a woman after oral ingestion of an ‘‘aroma liquid’’-type drug bought in a drug shop. This study identified and quantified PV9 (**32**) in the ‘‘aroma liquid’’ product as well as in antemortem and postmortem samples [40].

Majchrzak et al. [41] (2018) reported the first case of fatal poisoning with N-PP (**60**), a novel synthetic cathinone. This compound was identified in a white powder found at the scene and high concentrations were found in postmortem specimens collected from the autopsy [41]. Moreover, Pieprzyca et al. [42] (2018) reported two fatal poisoning cases in which PV8 (**31**) was detected and quantified in biological samples and found to be the cause of the deaths.

Adamowicz et al. [43] (2020) reported a fatal intoxication with α-PiHP (**48**). This substance was detected and quantified in all postmortem samples except in hair and was reported as the main cause of death, although 4-CMC, *N*-ethylhexedrone, benzoylecgonine and MDMA were also detected in some analyzed materials [43].

Currently, hundreds of synthetic cathinones have been identified and up to 250 new cathinone-related chemical entities are estimated to emerge every year [44]. The identification of these compounds and the implementation of a drug library with their structures and physicochemical and pharmacological properties are of great importance for chemists and toxicologists [14].

## 4. Toxicokinetic Properties

Substituted cathinones are more frequently administered orally or by nasal insufflation (snorting). Other pathways, such as rectal administration, intravenous or intramuscular injection, smoking or inhalation, are less common but have also been reported [17]. Moreover, some cases of insertion of synthetic cathinones into the eye (eyeballing) have been occasionally described [45].

In most cases, when compared to amphetamines, synthetic cathinones present a lower ability to cross the blood–brain barrier since the β-keto group increases their polarity. However, for the pyrrolidine derivatives, the presence of a pyrrolidine ring decreases their polarity increasing the permeability of the blood–brain barrier [3,46].

**Figure 10:**
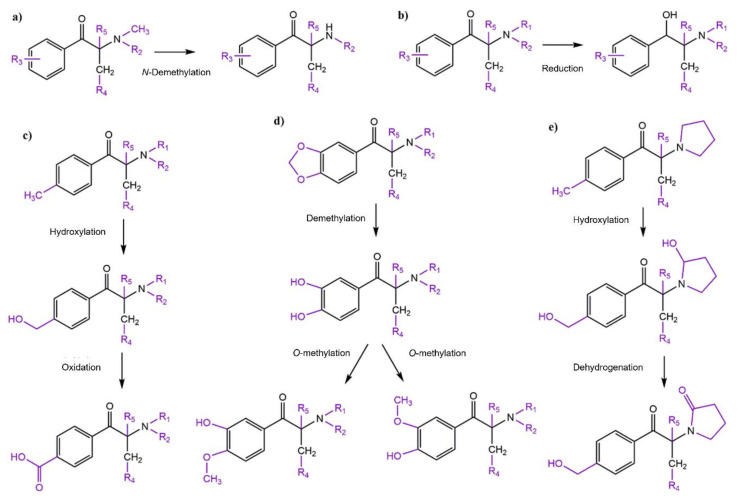
General metabolic pathways of synthetic cathinones: (**a**) main metabolic pathways of *N*-alkylated cathinones; (**b**) reduction of the β-keto moiety to an alcohol; (**c**) hydroxylation and further oxidation of the methyl substituent of the aromatic ring to a carboxylic acid; (**d**) metabolism of the 3,4-methylenedioxy ring; (**e**) metabolism of the pyrrolidinyl ring to a lactam.

Synthetic cathinones can be metabolized by several pathways from phase I and phase II reactions (Figure 10). 

Considering the phase I reactions, in a general way, each one of the four groups previously described (*N*-alkylated cathinones, pyrrolidinophenone cathinones, 3,4-methylenedioxy cathinones and mixed cathinones) present similar intragroup metabolic pathways [13]. 

For the first group, the *N*-alkylated cathinone derivatives, *N*-demethylation represents one of the main metabolic pathways (Figure 10a). However, since the β-keto group is shared among synthetic cathinones, for most of them, this moiety undergoes reduction to the corresponding alcohol (Figure 10b). Additionally, derivatives with a methyl group on the aromatic ring suffer hydroxylation of the methyl substituent, which can be further oxidized to the corresponding carboxylic acid (Figure 10c) [47,48,49]. The 3,4-methylenedioxy cathinones undergo demethylenation mediated by CYP2D6 and CYP2C19 followed by *O*-methylation mediated by catechol *O*-methyltransferase (COMT) (Figure 10d) [6,47]. 

Some general metabolic pathways found for the pyrrolidinyl ring of the pyrrolidinophenone derivatives are hydroxylation followed by dehydrogenation to the corresponding lactam (Figure 10e). However, differences can be found in the main metabolic pathways of the derivatives of this group depending on the alkyl chain length [14,47,48,49].

Lastly, the 3,4-methylenedioxypyrrolidinophenones share metabolic pathways with the corresponding methylenedioxy and pyrrolidinophenone derivatives [50]. 

The generated hydroxyl metabolites in the various metabolic pathways can undergo phase II metabolism (glucuronidation or sulfation) being the conjugates excreted in urine as well as the unmetabolized cathinones [6,47].

## 5. Mechanism of Action and Effects

**Figure 11:**
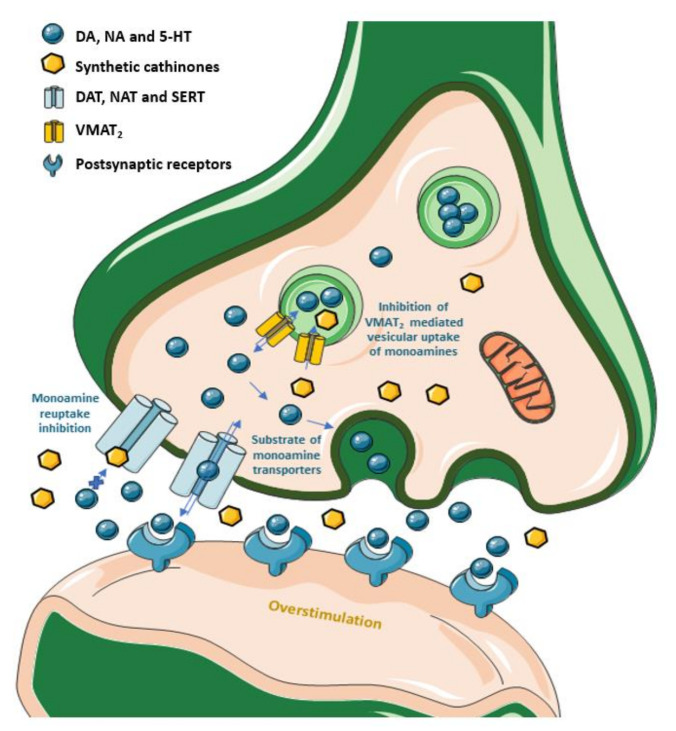
Modes of action of synthetic cathinones in the central nervous system. (DA: dopamine; NA: noradrenaline; 5-HT: 5-hydroxytryptamine (serotonin); DAT: dopamine transporters; NAT: noradrenaline transporters; SERT: serotonin transporters; VMAT_2_: vesicular monoamine transporter-2).

In a similar way to other illicit drugs, synthetic cathinones seem to display their psychostimulant properties due to interactions with membrane transporters for monoamines, such as noradrenaline transporters (NAT), serotonin transporters (SERT), and dopamine transporters (DAT) [4,6,20]. These interactions can occur through inhibition of the mononamine reuptake from the synaptic cleft (by binding to the NAT, SERT and/or DAT) and/or promotion of the release of monoamines (for instance, by inhibition of the vesicular monoamine transporter-2 (VMAT_2_)). Both mechanisms lead to an increase in extracellular concentrations of monoamines amplifying cell-to-cell monoamine signaling (Figure 11) [51,52]. The affinity of this interaction can, however, differ greatly between derivatives. Therefore, besides the previous classification based on the substitution pattern, synthetic cathinones can also be divided according to the type of interaction with monoamine transporters [4,6,20].

Firstly, some cathinones such as mephedrone (**9**), naphyrone (**21**) and methylone (**24**) have similar chemical structures to cocaine or/and MDMA, being, for that reason, designated as cocaine-MDMA-mixed synthetic cathinones. As cocaine, these cathinones can inhibit monoamine uptake in a nonselective way presenting more affinity to DAT than SERT. Additionally, except naphyrone (**21**), these derivatives promote the release of serotonin in a similar way to MDMA [4,14,20]. The second group, the methamphetamine-like synthetic cathinones, have a preferential reuptake inhibition of catecholamines and are dopamine releasers like methamphetamine. Cathinone (**1**), methcathinone (**8**) and 4-FMC (**16**) are part of this group. MDMA-like synthetic cathinones are the third group, which includes methedrone (**18**). This group is characterized by a great potency to inhibit NAT and SERT but low to DAT [4,14,20]. Finally, the fourth group is designated as pyrovalerone-cathinones since the synthetic cathinones that compose this group, such as MDPBP (**27**) and MDPV (**28**), present similar properties to pyrovalerone. They have great selectivity and potency to inhibit catecholamine uptake but do not promote the release of monoamines [4,20].

Synthetic cathinones are widely consumed for some of their effects such as euphoria, heightened senses and sensory perception, promotion of sociability, enhanced energy, mental stimulation, openness, empathic connection, decreased inhibition and increased libido [17]. However, along with all these effects, some other negative effects have also been reported by users. Since these compounds are simultaneously consumed with other drugs and their users might sometimes be clueless about which drug they have taken, it might be hard to relate the effects directly to synthetic cathinones [53]. Misunderstanding of the potency of these drugs can result in death, with overdose and suicide being the two most common causes (due to the psychological effects of the drug, such as loss of impulse control) [54].

Frequent adverse effects of synthetic cathinones are consistent with a sympathomimetic syndrome whose symptoms include delusions, hallucinations, paranoia, tachycardia, hypertension, abdominal pain, hyperthermia, dizziness, tremors, rhabdomyolysis and kidney damage [55]. Moreover, the use of high doses of synthetic cathinones can induce tolerance, dependence, craving and withdrawal syndrome after abrupt cessation. This syndrome might include symptoms such as sleep disorders, fatigue, depression, anxiety and craving [46].

## 6. Enantioselectivity Studies

Synthetic cathinones are chiral molecules meaning that they can exist in two enantiomeric forms that, consequently, can differ in their biological and toxicological properties [17]. Although synthetic cathinones are widely studied, few studies about enantioselectivity have been performed [56]. Nonetheless, the number of available studies has been growing and enantioselectivity was found in some cases. Relevant examples of enantioselectivity include a study by Glennon et al. [57] with the enantiomers of methcathinone (**8**), in which the *S*-enantiomer showed higher stimulating effects in the central nervous system than the *R*-enantiomer. 

**Figure 12:**
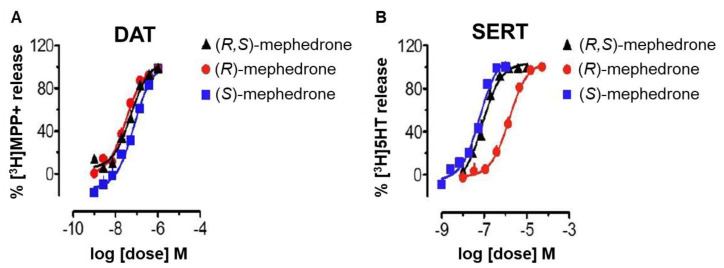
Effects of the enantiomers and racemate of mephedrone on monoamine release via DAT and SERT [58]. (**A**) DAT: dopamine transporters; MPP^+^: 1-methyl-4-phenylpyridinium (substrate for DAT); (**B**) SERT: serotonin transporters; 5HT: 5-hydroxytryptamine (serotonin).

Additionally, a study about the neurochemical effects of the enantiomers of mephedrone (**9**) in rats was performed by Gregg et al. [58], in which the enantiomers displayed some differences (Figure 12); while the *S*-enantiomer presented a higher serotonergic profile, the *R*-enantiomer showed a dopaminergic profile with locomotor activity and rewarding properties, suggesting higher addiction potential. Moreover, the *R*-enantiomer demonstrated less potency to serotonin transporters than the *S*-enantiomer or the racemate, resulting in lower release of serotonin [58].

**Figure 13:**
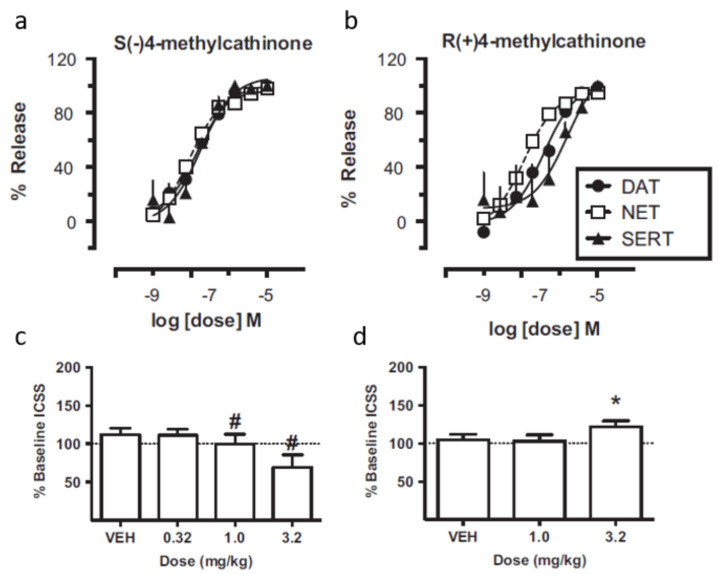
Effects of *S*-(−)4-methylcathinone and *R*-(+)-4methylcathinone on monoamine release via DAT, SERT and NET ((**a**) and (**b**), respectively) and on ICSS (**c**) and (**d**), respectively) [59]. DAT: dopamine transporters; NET: norepinephrine transporters; SERT: serotonin transporters; ICSS: intracranial self-stimulation. * indicate significant increases and # indicated significant decreases in ICSS rates relative to vehicle for at least one stimulation frequency as determined by analysis of frequency–rate curves in panels (**a**,**b**).

For the enantiomers of 4-methylcathinone (nor-mephedrone), the monoamine release and behavioral effects in rats through the response of electrical brain stimulation by an intracranial self-stimulation (ICSS) procedure were evaluated by Hutsell et al. [59] (Figure 13). In both in vitro and in vivo assays, the *S*-enantiomer showed higher potency than the *R*-enantiomer. For the in vitro assay, the *S*-enantiomer was able to promote monoamine release to a higher extent. However, the abuse potential of monoamine releasers seems to be related to their DAT vs SERT selectivity, meaning that compounds with higher selectivity to DAT present higher abuse potential than non-selective or SERT-selective compounds. In this study, the *R*-enantiomer displayed a higher DAT vs SERT than the *S*-enantiomer, meaning a higher abuse potential. Furthermore, the two enantiomers displayed qualitatively different effects in the ICSS behavioral study. The *R*-enantiomer facilitated ICSS while the *S*-enantiomer depressed it [59].

Similar studies were performed for the enantiomers of MDPV (**28**) by Kolanos et al. [60]. The *S*-(+)-enantiomer was found to be the most potent one, displaying a greater potency as a reuptake inhibitor of the monoamine transporters of dopamine and norepinephrine and facilitation of ICSS. On the other hand, the *R*-(−)-enantiomer was unsuccessful to change the ICSS [60]. Moreover, in another study, Gannon et al. [61] showed that the *S*-(+)-enantiomer is predominantly, if not entirely, responsible for the effects of the racemate on locomotor activity and core temperature. Silva et al. [62] evaluated the hepatotoxicity in vitro for both enantiomers in primary cultures of rat hepatocytes. In this case, no enantioselectivity was found. 

Recent examples, include the reinforcing effects of MDPV (**28**) and α-PVP (**22**) enantiomers, studied by Gannon et al. [63] in rats, to compare their potency and effectiveness. Although the enantiomers of both synthetic cathinones were found to be highly effective reinforcers, the *S*-enantiomers displayed greater potency than the *R*-enantiomers [63]. 

Since the *S*-enantiomer of mephedrone (**9**) was previously found to be a potent serotonin releaser with no significant rewarding effects when compared to the *R*-enantiomer, Philogene-Khalid et al. [64] performed a study of its potential ability to reduce anxiety and depression-like effects from withdrawal following chronic cocaine or MDPV abuse. As anticipated, this study found that *S*-enantiomer, at doses with no rewarding effects, can reduce withdrawal symptoms [64]. The same research group also investigated rewarding and reinforcing properties of the enantiomers of mephedrone (**9**). The results suggested that *R*-enantiomer was mainly responsible for these properties in the racemate [65]. 

**Figure 14:**
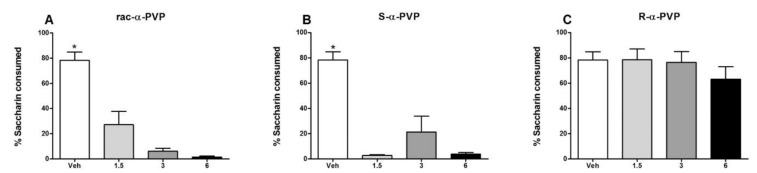
Avoidance tests with racemic α-PVP (**A**), S-α-PVP (**B**) and R-α-PVP (**C**). * Significantly different from 1.5, 3, and 5 [66].

In another study, Nelson et al. [66] assessed the contribution of the enantiomers of α-PVP (**22**) to the aversive effects of this synthetic cathinone using a conditioned taste avoidance (Figure 14). For this, a saccharin solution was associated with α-PVP. The racemate and *S*-enantiomer showed avoidance, while for the *R*-enantiomer no avoidance was observed. Moreover, it was found that the racemate displayed a greater avoidance than the additive effects of the enantiomers, suggesting that the *R*-enantiomer interacts synergistically with the *S*-enantiomer in the racemate [66].

More recently, the enantioselectivity of phase-I metabolites of mephedrone (**9**), nor-mephedrone, 4-hydroxytolyl-mephedrone (4-OH-mephedrone) and dihydro-mephedrone was evaluated by Mayer et al. [67]. All the enantiomers were found to be inhibitors of monoamine transporters; however, enantioselectivity was observed at the SERT inhibition effect, with the *S*-enantiomers of nor-mephedrone and 4-OH-mephedrone being more potent than the *R*-enantiomers. Urine sample analysis also found that the *S*-enantiomer of nor-mephedrone is the predominant form [67]. 

Schindler et al. [68] investigated the neurochemical, behavioral and cardiovascular effects of α-PVP (**22**) enantiomers in rats. Racemic α-PVP was able to inhibit dopamine and norepinephrine uptake, increase extracellular dopamine concentrations in the *nucleus accumbens*, increase locomotor activity, blood pressure and heart rate. It was found that the *S*-enantiomer is most likely to be responsible for these effects, since it was found to be 30-fold more potent than the *R*-enantiomer [68].

**Figure 15:**
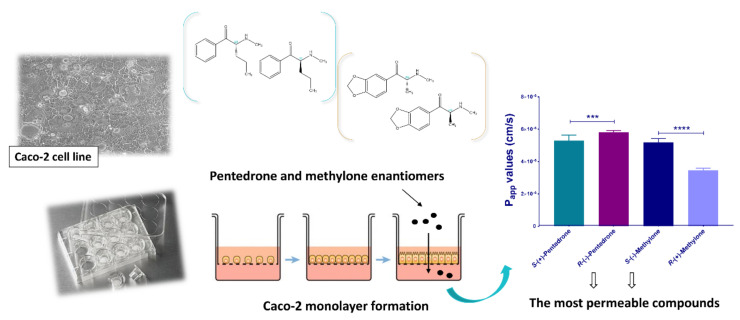
Enantioselectivity studies on the absorption of methylone (**24**) and pentedrone (**14**) using Caco-2 cell line. *** *p* < 0.001, **** *p* < 0.0001.

To evaluate the influence of chirality on the permeability across the gastrointestinal tract, Silva et al. [69] performed an in vitro study with the enantiomers of pentedrone (**14**) and methylone (**24**) (Figure 15) using the Caco-2 cell line. In this study, enantioselectivity was observed for both synthetic cathinones, with the *R*-(−)-enantiomer of pentedrone and the *S*-(−)-enantiomer of methylone being the most permeable compounds [69]. 

Davies et al. [70] studied the actions of the enantiomers of methcathinone (**8**) at monoamine transporters, discovering that they presented similar inhibition potencies at DAT and NET. At SERT, the *S*-enantiomer displayed a lower potency than at DAT and NET, while the *R*-enantiomer was practically inactive. Furthermore, in this study, an ICSS procedure to evaluate abuse-related drug effects in rats showed that the *S*-enantiomer presented almost twice the potency of the *R*-enantiomer [70].

**Figure 16:**
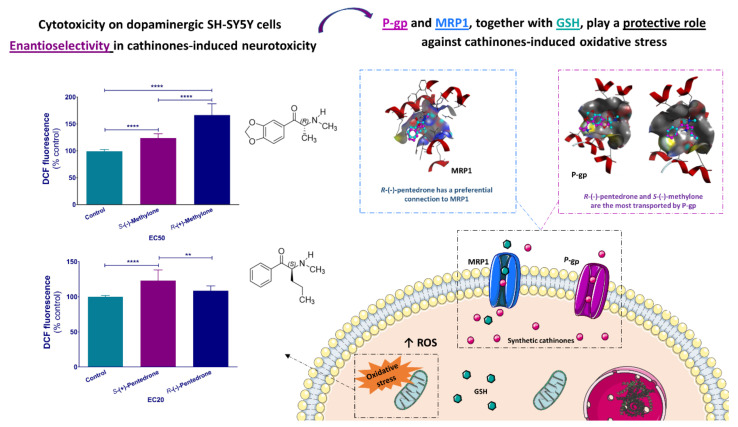
Enantioselectivity studies on neurocytotoxicity of methylone (**24**) and pentedrone (**14**) towards dopaminergic SH-SY5Y cells and the role of the efflux transporter multidrug-resistance-associated protein 1 (MRP1) and P-glycoprotein (P-gp). ** *p* < 0.01, **** *p* < 0.0001. (Reprint with permission from [71], Copyright (2021) Elsevier).

The most recent study about enantioselectivity of cathinone derivatives was performed by Silva et al. [71], which evaluated the enantioselective effect of pentedrone (**14**) and methylone (**24**) enantiomers in human neuronal cells. The results showed that the *S*-(+)-enantiomer of pentedrone and the *R*-(+)-enantiomer of methylone were the most oxidative and cytotoxic enantiomers (Figure 16). Additionally, *R*-(−)-pentedrone presented higher affinity to the efflux transporter multidrug-resistance-associated protein 1 (MRP1). It was also observed enantioselectivity in the binding to P-glycoprotein (P-gp) with *R*-(−)-pentedrone and *S*-(−)-methylone being the most transported enantiomers, which means a higher affinity to this efflux protein [71].

## 7. Enantiomeric Resolution 

In order to perform enantioselectivity studies, the enantiomers must be in their enantiomerically pure form [72]. A common way to achieve that is through the resolution of a racemate into the single enantiomers. The chiral separation of the enantiomers is important not only to further evaluate their enantioselectivity by testing the single enantiomers, but also to find out whether these drugs are sold as racemates or single enantiomers [73,74]. Determination of the enantiomeric composition of synthetic cathinones and other NPS may give information about the laboratory they come from, the starting material used for the synthesis, and even help the tracking of these compounds [75]. Thus, the development of analytical methods for the enantioseparation of synthetic cathinones is of great interest [76].

Several methods allow enantiomeric resolution; these can be divided into indirect and direct methods [77,78]. Indirect methods are based on the formation of diastereomers through derivatization of the enantiomers with an enantiomerically pure reagent via a covalent bond. The diastereomers are then separated under achiral conditions, by crystallization or chromatography methods, for example [79,80]. On the other hand, direct methods use a chiral selector present in the separation compartment. Chromatography is the most-used direct resolution method, in which the chiral selector can be a component of the stationary phase or an additive in the mobile phase [79]. The chiral selector binds preferentially one of the enantiomers, resulting in the formation of transient diastereomeric complexes with different stabilities and, consequently, different retention times. The less stable diastereomeric complex is eluted first [77]. The direct approach is frequently preferred over the indirect, since there is no need for previous derivatization, less sample manipulation is needed and the results are rapidly obtained after the separation [81].

Chromatographic enantioseparations by gas chromatography (GC) and liquid chromatography (LC) can be performed through either indirect or direct methods. For GC, an indirect approach is the most common since few chiral stationary phases (CSPs) are available. For LC, high-performance liquid chromatography (HPLC) using CSPs is the most-used method since there are several available CSPs. Moreover, this method can be coupled with different detection methods such as ultra-violet (UV)-visible (vis) absorption and mass spectrometry (MS), which is an advantage [82]. Thus, HPLC using CSPs is considered the most versatile and practical method, being used for both analytical and preparative purposes [83]. Nonetheless, ultra-high-performance liquid chromatography (UHPLC) has been gaining more attention, since it has higher selectivity, efficiency, and a shorter analysis time than HPLC and there are already available CSPs that can be adapted for this method [84,85].

Additionally, supercritical fluid chromatography (SFC), a hybrid of GC and LC, is another chromatographic method that can be used in direct chiral separation. Although this method allows a faster separation of the enantiomers than HPLC, it presents higher costs and more complex hardware and, as a result, only a few studies are reported [82]. Moreover, direct enantiomeric separation can also use capillary electromigration techniques such as capillary electrophoresis (CE) and capillary electrochromatography (CEC) [86,87,88]. These methods are based on electrophoretic phenomena for the movement of the enantiomers [79,89,90]. In CE, chiral selectors, such as cyclodextrins (CD) and their derivatives, are usually added to a running buffer [91,92]. For CEC, although the mobile phase is also driven by electroosmosis like in CE, the separation mechanism is based on the partition between the liquid and stationary phases, like in HPLC, making this technique a hybrid of CE and HPLC [82,93,94]. As will be shown, all these resolution techniques have been used for enantioseparation of cathinone derivatives. 

Schmid and Hagele [95] reviewed different techniques that have been developed for the enantiomeric separation of chiral NPS comprising drugs such as cathinones, amphetamines and ketamines. Similarly, Silva et al. [56] have focused their studies on the chiral separation of synthetic cathinones. 

Silva et al. [56] found 12 direct HPLC studies using UV detection and different types of CSPs [62,73,96,97,98,99,100,101]. Aboul-Enein and Serignese [96,97] developed two direct HPLC methods for the separation of the enantiomers of cathinone using protein-based and crown-ether CSPs. Wolrab et al. [98] performed the enantioseparation of 14 cathinone derivatives by HPLC using structurally different ion-exchange-type CSPs. Moreover, Silva et al. [62] successfully separated nine cathinone derivatives using a HPLC method with polysaccharide-based CSPs under normal phase elution conditions.

Additionally, three CE [101,102,103] and three CEC [73,104] methods were also described using a direct chiral separation. For instance, CE using CD derivatives as additives for the buffer was used by Merola et al. [102] to separate 12 cathinone derivatives. Ten cathinones were separated using β-CD with UV detection and the other two were separated using highly sulfated (HS)-γ-CD with MS detection [102].

Albals et al. [73] performed a comparative study between CEC, SFC and three LC modes: polar organic solvent chromatography (POSC), reversed-phase liquid chromatography (RPLC) and normal-phase liquid chromatography (NPLC). Four different polysaccharide-based CSPs were used for chiral separation of ten cathinone and amphetamine derivatives [73]. 

Besides that, as indirect chiral separations of synthetic cathinones, three GC-MS methods [74,75,76,101] were found using trifluoroacetyl-L-prolyl chloride (L-TPC) as a chiral derivatization agent, and lastly, one crystallization method for the chiral separation of MDPV [105].

Most studies were performed with solid samples of cathinones bought online, obtained from seized drugs, or some even synthesized in the laboratories, apart from one. We emphasize a study by Baciu et al. [103], which developed a method for the chiral separation of mephedrone (**9**) and MDPV (**28**) in human hair samples using CE combined in-line with solid-phase extraction (SPE). 

Recently, since 2018, many other enantioseparation studies of synthetic cathinones were performed which are compiled in Table 1.

**Figure 17:**
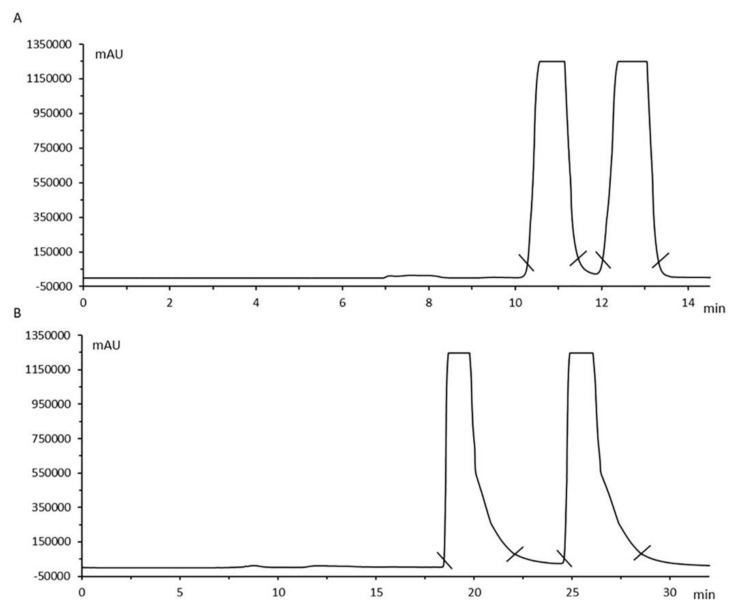
Chromatograms of the semipreparative enantioseparation of pentedrone (**A**) and methylone (**B**). (Reprint with permission from [111], Copyright (2018) Elsevier).

As shown in Table 1, recently, Alremeithi et al. [108] developed a highly sensitive and selective method for the separation of 14 cathinone derivatives in urine and plasma samples using GC-MS and L-TPC as a derivatization agent. Meetani et al. [113] were able to detect and quantify, for the first time, the enantiomers of 18 synthetic cathinones with tertiary amine structures in urine and plasma samples using a direct HPLC-UV method with amylose-based and cellulose-based CSPs. Moreover, Loganathan et al. [125] developed an indirect HPLC-MS/MS method using N_α_-(2,4-dinitro-5-fluorophenyl)-L-valinamide (DNFP-L-V) as a derivatization agent for the detection, resolution and quantitation of cathinone enantiomers in horse blood plasma and urine samples, which can be useful for equine anti-doping analysis [125]. Silva et al. [111] performed, for the first time, the enantioseparation on a semipreparative scale of the enantiomers of pentedrone (**14**) and methylone (**24**) by HPLC-UV using an amylose-based CSP (Figure 17).

**Figure 18:**
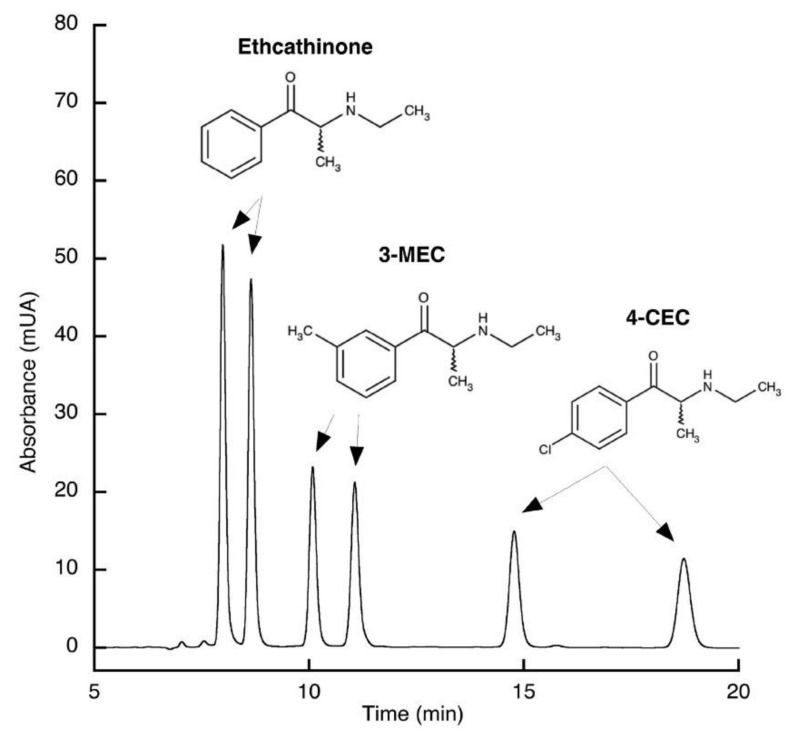
Chiral separation of three different cathinone derivatives (ethcathinone, 3-MEC and 4-CEC) by HPLC-UV [117].

In another study, Fu et al. [116] reported a direct method for the chiral separation of cathinones in environmental water samples using LC coupled with high-resolution mass spectrometry (HRMS) [116]. Many of the most recent methods were able to successfully separate the enantiomers of a vast number of cathinones derivatives. For instance, Hagele et al. [117] used an HPLC-UV method to separate the enantiomers of 39 cathinone derivatives which included ethcathinone, 3-MEC and 4-CEC (Figure 18).

Moreover, Kadkhodaei et al. [109] developed a direct HPLC-UV method using a cellulose-based CSP, which had the ability to separate 47 synthetic cathinones. Hagele et al. [112] used β-CD-assisted CE to separate 58 cathinone derivatives. Kadkhodaei et al. [119] separated 62 cathinone derivatives along with some other NPS with a direct HPLC-UV method using an amylose-based CSP. Additionally, these studies found that all the analyzed NPS were purchased as racemic compounds [109,112,119].

**Figure 19:**
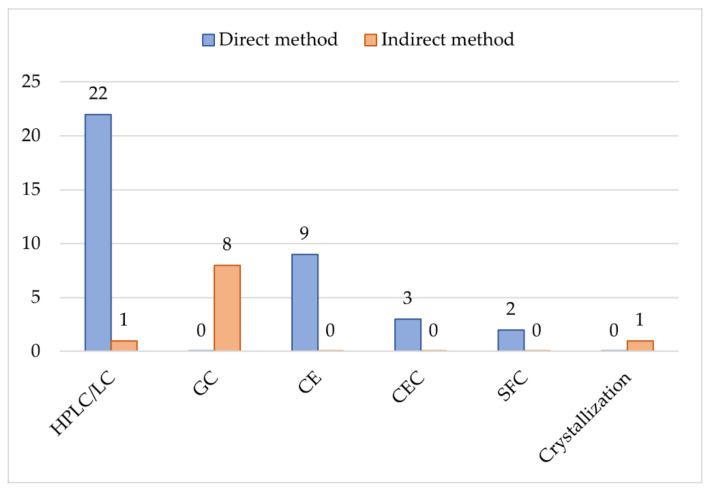
Methods used for the enantiomeric resolution of cathinones. HPLC: high-performance liquid chromatography; LC: liquid chromatography; GC: gas chromatography; CE: capillary electrophoresis; CEC: capillary electrochromatography; SFC: super critical fluid chromatography.

Figure 19 summarizes all studies previously compiled by Silva et al. [56] in addition to more recent studies compiled in Table 1, where it is possible to observe that direct methods are preferred over indirect methods. Besides that, HPLC is undoubtedly the most used technique. Only one of the 23 HPLC/LC studies reported an indirect chiral separation that used a derivatization step. Moreover, with exception of one study that used a ß-CD derivative as a chiral addictive for the mobile phase, all the studies used CSPs. Regarding the detection mode, most HPLC/LC methods used UV-absorption detection. Only three studies used MS detection. For UV detection, mobile phases generally contain non-volatile buffers, while for MS detection, volatile buffers are necessary. Furthermore, since MS detection needs the formation of ions, the mobile phase should be used to create charged analytes. Thus, the mobile phase pH and the pKa of the analyte are important parameters for this detection. The selection of the pH of the mobile phase can increase sensitivity [127].

For GC-MS, all the methods mentioned were indirect chiral separations as well as the crystallization method previously mentioned. CE, CEC and SFC were all performed as a direct chiral separation. However, while for CE, chiral additives were added to the BGE to allow an enantiomeric separation, for the CEC and SFC techniques, CSPs were used. Additionally, NPLC and RPLC modes were the most used.

**Figure 20:**
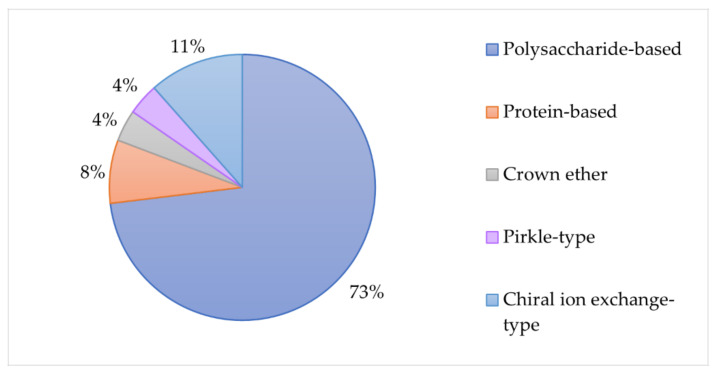
Types of CSPs used for the enantiomeric resolution of cathinones.

Furthermore, the pie chart in Figure 20 shows the type of CSPs used in all the HPLC/LC, CEC and SFC methods described above. Clearly, polysaccharide-based CSPs are the preferred type for the enantioseparation of cathinones since they were chosen in most of the studies. Only 27% of the methods used other type of CSPs. 

## 8. Conclusions

This review presents an up-to-date report of synthetic cathinones described for the first time since 2014, which include more than 50 derivatives. Synthetic cathinones are still widely abused and novel derivatives keep emerging every year with unknown chemical and biological properties, some of them after minor chemical structure modifications. Consequently, there is still a long way to go to achieve the identification and characterization of all new synthetic cathinones, particularly the properties related to chirality. 

Furthermore, this review highlights the relevance of the stereochemistry of synthetic cathinones, which is often overlooked, providing a compilation of the most recent developments in enantioselectivity studies and enantioresolution methods, which will be very useful for everyone working in this research field, and affording innovative perspectives on this topic. Most of the enantioselectivity studies evidenced that the enantiomers of cathinone derivatives displayed different toxicokinetic and/or toxicodynamic properties. These studies are important to determine which enantiomer is responsible for the main biological or toxicological effects and/or potency, presenting a crucial role in cases of cathinone abuse. 

Regarding the enantiomeric resolution methods, it was found that HPLC using polysaccharide-based CSPs was the most-used method for the enantioseparation of synthetic cathinones. Even if the number of studies considering stereochemical issues in both biological/toxicological activities and enantioresolution analysis has been growing, it is crucial to go deeper into research regarding the enantioselectivity of these drugs of abuse as the consumption of cathinones continues to increase.

## Figures and Tables

**Table 1:** Development of analytical methods for enantioresolution of synthetic cathinones from 2018 to 2021.

Analyte	Sample	Method	Analytical conditions	Ref.
3-FMC; 4-FEC; Ethcathinone; Buphedrone; 3-MMC; Pentedrone; 4-Methylbuphedrone; 3,4-DMMC; Methedrone; 2,3-MDMC; Eutylone; Pentylone	Urine and plasma	GC-MS (indirect method)	Achiral stationary phase: HP-5MS capillary column Derivatization with L-TPC	[106]
2-AIMP; bk-iVP; 4-BMC; 4-CMC; 5-DBFPV; DL-4662; 4-FMC; 4F-PV8; Methedrone; 3-MeOMC; 3-MEC; 4-MEC; 2-MMC; 3-MMC; 4-MMC; 5-PPDi; α-PVP; 4-MeO-α-PVP; TH-PVP	Solid	HPLC-UV (direct method)	Polar organic mode CSP: Lux^®^ Cellulose-2 column Mobile phase: ACN/IPA/DEA/FA (95:5:0.1:0.1) Flow rate: 1 mL/min UV detection: 254 nm	[107]
4-FMC; 4-FEC; Nor-mephedrone; Buphedrone; 3-MMC; 3-Methylbuphedrone; 4-Methylbuphedrone; 3-EMC; 3-EEC; 4-EEC; 3,4-DMEC; 2,3-MDMC; Butylone; Pentylone	Urine and plasma	GC-MS (indirect method)	Achiral stationary phase: HP-5MS Ultra-Inert capillary column Derivatization with L-TPC Flow rate: 0.8 mL/min	[108]
4-MMC; 3-MMC; 2-MMC; 3,4-DMMC; 4-MeOMC; 3-MeOMC; 3-CMC; 4-CMC; 4-EMC; Mexedrone; 4-FMC; 3-FMC; 2-FMC; 4-BMC; Buphedrone; 4-Methylbuphedrone; Pentedrone; 3-CEC; 4-CEC; *N*-Ethyl-Buphedrone; *N*-Ethyl-Hexedrone; Amfepramone; 4-MEC; 3-MEC; Methylone; Dimethylone; Butylone; *N,N*-Dimethylbutylone; Pentylone; Ethylone; 5-ME; *N*-Ethylpentylone; MDPV; MD-PHP; bk-IVP; 5-DBFPV; DOMC; 5-PPDI; TH-PVP; 4-MC; α-PPP; M-PPP; 4-MPrC; 4-MeO-α-PVP; 4-Cl-PVP; Naphyrone	Hydrochloride salts	HPLC-UV (direct method)	NPLC mode CSP: Lux^®^ i-cellulose-5 column Mobile phase :Hex/IPA/DEA (95:5:0.1), Flow rate: 1 mL/min UV detection: 254 nm	[109]
Methcathinone; 4-MMC and 3-MMC	Solid	CE (direct method)	BGE: phosphate buffer I (H3PO4/NaH2PO4, pH 3.0), acetic buffer (CH3COOH/CH3COONa, pH 5.0), and phosphate buffer II (NaH2PO4/Na2HPO4, pH 8.7), all of 50 mM ionic strength with different CD additives DAD: set at 200 nm	[110]
Pentedrone	Powder	HPLC-UV (direct method)	NPLC mode CSP: Chiralpak^®^ AS-H column Mobile phase: Hex/IPA (97:3) Flow rate: 2 mL/min UV detection: 254 nm	[111]
Methylone	Powder	HPLC-UV (direct method)	NPLC mode CSP: Chiralpak^®^ AS-H column Mobile phase: Hex/IPA (85:15, *v*/*v*) Flow rate: 2 mL/min UV detection: 254 nm	[111]
4-MC; 2-MMC; 3-MMC; 4-MMC; 3,4-DMMC; 3-MeO-MC; Methedrone; 3-CMC; 4-CMC; 4-EMC; Mexedrone; 2-FMC; 3-FMC; 4-FMC; 4-BMC; Buphedrone; 4-Methylbuphedrone; Pentedrone; Amfepramone; 3-CEC; 4-CEC; DL-4662; *N*-Ethylhexedrone; 3-MEC; 4-MEC; Bupropione; 4-MPD; *N*-Ethylbuphedrone; *N*-Ethylpentedrone; Ethylone; *N*-Ethylpentylone; 5-ME; bk-Ivp; 5-DBFPV; DOMC; 5-PPDi; 4-MBC; Methylone; 2-AIMP; Dimethylone; Butylone; *N*-Benzylnorbutylone; *N,N*-Dimethylbutylone; Pentylone; PV8; 4-F-PV8; α-PVP; 4-Cl-PVP; 4F-PVP; 4-MeO-α-PVP; PV9; α-PPP; M-PPP; α-PIHP; 4F-PHP; Naphyrone; MDPV; MDPHP	Solid	CE (direct method)	BGE: 10 mM of a β-CD derivative, 10 mM sodium phosphate adjusted with diluted phosphoric acid (pH 2.5) DAD: set at 209 nm	[112]
Dimethylone; α-PPP; *N,N*-DMC; 2-Methyl-α-PPP; 4-Ethyl-*N,N*-DMC; 3-Methyl-α-PPP; 3,4-MD-α-PPP; 4′-MeO-α-PPP; 4′-Methyl-α-PHP; Diethylcathinone; 4-Methyl PBP; α-PVP; α-PBP; 4′-Methyl-α-PPP; 3-Methyl PBP; 3,4-MDPBP; *N*-Ethyl-N-Methylcathinone; 2-Methyl PBP; 4-Meo-*N,N*-DMC	Blood and urine	HPLC-UV (direct method)	CSP: Astec^®^ Cellulose DMP column Mobile phase: Hex/IPA/TEA (99.0:1.0:0.1) Flow rate: 0.5 mL/min UV detection: 270 nm	[113]
Dimethylone; *N,N*-DMC; 2-Methyl-α-PPP; 4-Ethyl-*N,N*-DMC; 4′-MeO-α-PPP; 3,4-MDPBP; 2-Methyl PBP	Blood and urine	HPLC-UV (direct method)	Direct chiral separation: CSP: Amylose-based Chiralpak^®^ AS-H Mobile phase: Hex/IPA/TEA (99.0:1.0:0.1) Flow rate: 0.5 mL/min UV detection: 270 nm	[113]
MDPV; Mephedrone; Methylephedrine	Urine	SPE-CE (direct method)	BGE: aqueous solution of 70 mM of monosodium phosphate, adjusted to pH 2.5 with concentrated phosphoric acid, containing 8 mM 2-hydroxypropil-β-CD and 5 mM β-CD DAD: set at 200 nm	[114]
Cathinone	*Catha edulis*	GC-MS (indirect method)	Achiral stationary phase: HP-5 MSI capillary column Derivatization with MCF Flow rate: 1 mL/min	[115]
Mephedrone; Butylone: Flephedrone; Methylone; Methedrone	River water	LC-HRMS (direct method)	RPLC mode CSP: Chiralpak^®^ CBH column Mobile phase: 1 mM ammonium acetate buffer/MeOH (98:2) Flow rate: 0.4 mL/min	[116]
Nor-Mephedrone; 3-MMC; 4-MMC; 3,4-DMMC; 3-MeO-MC; Methedrone; 3-CMC; 4-CMC; 4-EMC; Mexedrone; 2-FMC; 3-FMC; 4-FMC; 4-BMC; Buphedrone; 4-Methylbuphedrone; Pentedrone; 3-CEC; 4-CEC; DL-4662; 3-MEC; 4-MEC; Ethcathinone; 4-MPD; *N*-ethylbuphedrone; *N*-ethylpentedrone; 4-ethylcathinone; Methylone; 2-AIMP; Dimethylone; Butylone; *N,N*-dimethylbutylone; Pentylone; Ethylone; 5-ME; bk-iVP; 5-DBFPV; DOMC; 5-PPDi	Hydrochloride salts	HPLC-UV (direct method)	CSP: Phenomenex Lux^®^ AMP Mobile phase: ammonium bicarbonate (5 mM) adjusted to pH 11.3 with conc. ammonium hydroxide/ ACN (70:30) Flow rate: 0.5 mL/min UV detection: 230 nm	[117]
4-MC; 4-MMC; 3-MMC; 3,4-DMMC; 3-CMC; 4-CMC; 4-EMC; 4-FMC; 4-BMC; Buphedrone; 4-Methylbuphedrone; Ethcathinone; 4-EEC; 3-CEC; 4-CEC; *N*-Ethylbuphedrone; *N*-Ethylpentedrone; DL-4662; 3-MEC; 4-MEC; *N*-Propcathinone; 4-MPC; 4-CPRC; Dimethylone; 2-AIMP; Butylone; Ethylone; 5-ME; *N*-Ethylpentylone; 4-MBC; bk-IVP; DOMC; 4-CDC	Hydrochloride salts	HPLC-UV (direct method)	NPLC mode CSP: Trefoil^®^ CEL1 column with cellulose tris-(3,5-dimethylphenyl-carbamate) Mobile phase: Hex/ButOH/DEA (100:0.3:0.2) Flow rate: 1 mL/min UV detection: 230 nm	[118]
4-MMC; 3-MMC; 2-MMC; Methedrone; 3-MeoMC; 4-CMC; 4-BMC; 4-FMC; 4-EMC; Mexedrone; Buphedrone; 4-Methylbuphedrone; Pentedrone; 3-CEC; 4-CEC; 4 MPD; *N*-Ethyl-pentedrone; DL-4662; 4-EEC; 4-MPC; 4-CPRC; 4-F-PVP; 4 M-PHP; *N*-Ethylpentylone; MDPV and TH-PVP	Solid	HPLC-UV (direct method)	NPLC mode CSP: Lux^®^ i-Amylose-1 column Mobile phase: Hex/IPA/DEA (90:10:0.1) Flow rate: 1 mL/min UV detection: 254 nm	[119]
4-MC; 3-CMC; 2-FMC; 3-FMC: 3,4-DMMC; *N*-ethyl-buphedrone; *N*-ethyl-hexedrone; Amfepramone; 3-MEC; 4-MEC; Ethcathinone; 4-ClC; 4-Chlorbutcathinone; α-PPP; M-PPP; α-PVP; 4-Cl-PVP; 4-MPrC; 4-MeO-α-PVP; Naphyrone; Methylone; Dimethylone; 2-AIMP; Butylone; *N,N*-Dimethylbutylone; Pentylone; 5-ME; Ethylone; MD-PHP; bk-iVP; 5-DBFPV; DOMC; 5-PPDI; 5-BPDI; 4-MBC	Solid	HPLC-UV (direct method)	NPLC mode CSP: Lux^®^ i-Amylose-1 column Mobile phase: Hex/IPA/DEA (99:10:0.1) Flow rate: 1 mL/min UV detection: 254 nm	[119]
4-MMC; 3,4-DMMC; 4-EMC; 4-MEC; 4-Methylbuphedrone; Buphedrone; *N*-Ethylbuphedrone; Pentedrone; Pyrovalerone; bk-PMA; bk-PMMA; Methylone; Ethylone; Butylone; Pentylone; MDPV; MDPBP; Naphyrone; 4F-NEB; 4F-MABP; 2-FMC; 4-FMC; 4-CMC; 4-BMC	Solid	SFC-MS (direct method)	CSP: Chiralpak^®^ ZWIX (+) and Chiralpak^®^ ZWIX (−) Mobile phase: MeOH/H_2_O/FA (90:10:1) using a gradient elution method Flow rate: 1 mL/min	[120]
Methylone and ethylone	Crystals	LC- MS/MS (direct method)	RPLC mode CSP: Lux^®^ AMP polysaccharide-based chiral column Mobile phase: MeOH with a decreasing concentration gradient from 95% to 85% Flow rate: 0.48 mL/min	[121]
2-FMC; 2-FEC; Buphedrone; 3-MMC; 4-MEC; 3-MethylBP; 2,4-DMMC; 4-Methyl-α-ethylaminobutiophenone; 3,4-DMEC; 4-BMC; Butylone	Urine	GC-NCI-MS/MS (indirect method)	Achiral stationary phase: Agilent Ultra Inert capillary column Derivatization with MCF Flow rate: 1 mL/min	[122]
Mephedrone; Methylone; 4-Methylephedrine; MDPV	Urine	EKS-CE (direct method)	BGE: 70 mM of monosodium phosphate, 8 mM of 2-hydroxypropyl β-CD and 5 mM of β-CD (adjusted to pH 2.5 with concentrated phosphoric acid) DAD: set at 220 nm	[123]
Mephedrone and its metabolites	Hydrochloride salts	CE (direct method)	BGE: 50 mmol/L Phosphate buffer; pH 2.75; 7.5 mmol/L CM-β-CD DAD: set at 258; 236 or 214 nm	[124]
Cathinone	Horse plasma and urine	HPLC-MS/MS (indirect method)	RPLC mode Achiral stationary phase: fused core HALO-C18 column Mobile phase: 5 mM ammonium formate/0.1 % FA in H_2_O/ACN, in linear gradient Derivatization with DNFP-L-V Flow rate: 0.3 mL/min	[125]
MDPV	Urine	SPE-CE-MS (direct method)	BGE: 10 mM ammonium acetate aqueous solution (pH 7) with 0.5% (*m*/*v*) of sulphated-α-CD Sheath liquid: IPA/H_2_O/FA 60:40:0.25 (*v*/*v*) Flow rate: 3.3 μL/min	[126]

ACN: Acetonitrile; BGE: Background electrolyte; ButOH: Butanol; CBH: Cellobiohydrolase I; CD: Cyclodextrin; CE: Capillary electrophoresis; CEC: Capillary electrochromatography; CM-β-CD: Carboxymethyl-β-cyclodextrin; CSP: Chiral stationary phase; DAD: Diode array detection; DEA: Diethylamine; DNFP-L-V: *N*α-(2,4-Dinitro-5-fluorophenyl)-L-valinamide; EKS: Electrokinetic supercharging; FA: Formic acid; GC: Gas chromatography; Hex: Hexane; HPLC: High-performance liquid chromatography; HRMS: High resolution mass spectrometry; IPA: Isopropyl alcohol; L-TPC: Trifluoroacetyl-L-prolyl chloride; MCF: (1*R*)-(–)-Menthylchloroformate; MeOH: Methanol; MS: Mass spectrometry; NCI: Negative ion chemical ionization; NPLC: Normal-phase liquid chromatography; RPLC: Reversed-phase liquid chromatography; SFC: Super critical fluid chromatography; SPE: Solid phase extraction; TEA: Triethylamine; UV: Ultra-violet.

## Data Availability

Not applicable.

## Abbreviations

2:4-DMEC	2,4-Dimethylethcathinone
2,4-DMMC	2,4-Dimethylmethcathinone
2,4-DMPPP	2,4-Dimethyl-α-pyrrolidinopropiophenone
2,4-iso-DMC	2,4-Dimethylisocathinone
3,4-Dimethoxy-α-PVP	3,4-Dimethoxy-α-pyrrolidinopentiophenone
3-FMC	3-Fluoromethcathinone
4 F-PBP	4′-Fluoro-α-pyrrolidinobutyrophenone
4-BEC	4-Bromoethcathinone
4-Br-PPP	4-Bromo-α-pyrrolidinopropiophenone
4-Br-PVP	1-(4-Bromophenyl)-2-(pyrrolidin-1-yl)pentan-1-one
4-Cl-PPP	1-(4-Chlorophenyl)-2-(pyrrolidin-1-yl)propan-1-one
4-Cl-α-EAPP	1-(4-Chlorophenyl)-2-(ethylamino)pentan-1-one
4-Cl-α-PHP	1-(4-Chlorophenyl)-2-(pyrrolidin-1-yl)hexan-1-one]
4-FMC	4-Fluoromethcathinone
4F-PHP	1-(4-Fluorophenyl)-2-(pyrrolidin-1-yl)hexanone
4-F-α-PHPP	4-Fluoro-α-pyrrolidinoheptanophenone
4-F-α-PVP	4-Fluoro-α-pyrrolidinopentiophenone
4-MEC	4-Methyl-*N*-ethylcathinone
4-Methoxy-α-PHPP	4-methoxy-α-pyrrolidinoheptanophenone
4-Methoxy-α-POP	4-Methoxy-α-pyrrolidinooctanophenone
4-Methoxy-α-PVP	4-Methoxy-α-pyrrolidinovalerophenone)
4-MPD	4-Methylpentedrone
5-BPDI	1-(2,3-Dihydro-1H-inden-5-yl)-2-(pyrrolidin-1-yl)hexan-1-one
5-HT	5-Hydroxytryptamine
5-PPDI	1-(2,3-Dihydro-1*H*-inden-5-yl)-2-(pyrrolidin-1-yl)butan-1-one
ACN	Acetonitrile
AGP	α_1_-Acid glycoprotein
BGE	Background electrolyte
bk-MDA	2-Amino-1-(benzo[d][1,3]dioxol-5-yl)propan-1-one
bk-PDP	1-(1,3-Benzodioxol-5-yl)-2-(ethylamino)-1-pentanone
BMAPN	2-(Methylamino)-1-(naphthalen-2-yl) propan-1-one
BTA	2′-Bromotartranilic acid
ButOH	Butanol
CBH	Cellobiohydrolase I
CD	Cyclodextrin
CE	Capillary electrophoresis
CEC	Capillary electrochromatography
COMT	Catechol *O*-methyltransferase
CSP	Chiral stationary phase
DAD	Diode array detection
DAT	Dopamine transporters
DEA	Diethylamine
DEB	*N,N*-Diethylbuphedrone
DEP	*N,N*: Diethylpentedrone
DMB	*N,N*-Dimethylbuphedrone
DMP	*N,N*-Dimethylpentedrone
DNFP-L-V	*N*α-(2,4-Dinitro-5-fluorophenyl)-L-valinamide
EKS	Electrokinetic supercharging
EMCDDA	European Monitoring Centre for Drugs and Drug Addiction
EPH	Ephedrone or Methcathinone
EtOH	Ethanol
FA	Formic acid
GC	Gas chromatography
Hex	Hexane
Hexedrone	α-Methylaminohexanophenone
HPLC	High performance liquid chromatography
HRMS	High resolution mass spectrometry
HSA	Human serum albumin
ICSS	Intracranial self-stimulation
IPA	Isopropyl alcohol
LC	Liquid chromatography
L-TPC	Trifluoroacetyl-L-prolyl chloride
MCF	(1*R*)-(–)-Menthylchloroformate
MDMA	3,4-Methylenedioxymethamphetamine
MDPV	3,4-Methylenedioxypyrovalerone
MeOH	Methanol
MEPH	Mephedrone
MPHP	4-Methyl-α-pyrrolidinohexanophenone
MPP	2-Methyl-1-phenyl-2-(pyrrolidin-1-yl)propan-1-one
MPP^+^	1-Methyl-4-phenylpyridinium
MRP1	Multidrug resistance associated protein 1
MS	Mass spectrometry
NAT	Noradrenaline transporters
NCI	Negative ion chemical ionization
NET	Norepinephrine transporters
NPLC	Normal-phase liquid chromatography
N-PP	α-Propyloaminopentiophenone
NPS	New psychoactive substances
P-gp	P-glycoprotein
POSC	Polar organic solvent chromatography
RPLC	Reversed-phase liquid chromatography
SERT	Serotonin transporters
SFC	Super critical fluid chromatography
SPE	Solid-phase extraction
TEA	Triethylamine
TFA	Trifluoroacetic acid
UHPLC	Ultra-high-performance liquid chromatography
UV	Ultra-violet
Vis	visible
VMAT_2_	Vesicular monoamine transporter-2
α-EAPP	α-Ethylaminopentiophenone
α-PBT	α-Pyrrolidinobutiothiophenone
α-PHP	α-Pyrrolidinohexanophenone
α-PHPP	α-Pyrrolidinoheptanophenone
α-PiHP	4-Methyl-1-phenyl-2-(pyrrolidin-1-yl)pentan-1-one
α-POP	α-Pyrrolidinooctanophenone
α-PVP	α-Pyrrolidinopentiophenone
α-PVT	α-Pyrrolidinopentiothiophenone
